# Investigation of the Impact of Living Costs on the Quality of Life of Patients With Moderate and Severe Traumatic Brain Injury

**DOI:** 10.7759/cureus.105807

**Published:** 2026-03-25

**Authors:** Dimitrios Cholevas, Eleni Theodosopoulou, Michael Kourakos, George Intas, Pantelis I Stergiannis

**Affiliations:** 1 Intermediate Care Unit, KAT General Hospital of Attica, Athens, GRC; 2 Faculty of Nursing, National and Kapodistrian University of Athens, Athens, GRC; 3 Nursing Department, School of Health Sciences, University of Ioannina, Ioannina, GRC; 4 Nursing, General Hospital of Nikaia, Athens, GRC

**Keywords:** economic burden, glasgow coma scale, quality of life, rehabilitation cost, traumatic brain injury

## Abstract

Introduction: Traumatic brain injury (TBI) significantly affects patients’ quality of life (QoL) through long-term physical, cognitive, and psychological consequences. Improved survival rates have increased the public health importance of understanding the economic burden of TBI and its impact on QoL. This study aimed to investigate the association between rehabilitation-related living costs and QoL in patients with moderate and severe TBI.

Methods: A prospective longitudinal observational study was conducted among adults with moderate and severe TBI hospitalized at General Hospital of Attica “KAT,” Athens, Greece. QoL was assessed using the Short Form-36 questionnaire, while household economic burden was evaluated using a structured cost questionnaire. Data were collected at discharge, at six months, and at 12 months post-discharge.

Results: The study included 100 patients (mean age 40.72 ± 10.9 years), of whom 64% had moderate and 36% severe injury. QoL was significantly lower in severe cases at all time points (p < 0.001). Monthly family income showed a positive association with QoL, particularly at six months. The mean rehabilitation cost during the first six months was €4,212.60 ± 307.40. The total 12-month household economic burden reached €9,092.00 ± 5,634.15. Increased economic burden was significantly associated with lower QoL, especially in mental health and social functioning dimensions.

Conclusion: TBI imposes a substantial long-term economic and QoL burden. Injury severity and financial resources strongly influence recovery. Integrated health and social support policies are essential to improve long-term outcomes.

## Introduction

Traumatic brain injury (TBI) disrupts normal brain function and may result from external mechanical forces such as falls, motor vehicle accidents, or violent incidents. TBI represents a major global health concern and remains one of the leading causes of injury-related morbidity and mortality worldwide. Epidemiological studies indicate that TBI imposes a substantial burden on healthcare systems and society due to long-term disability and rehabilitation needs [1,2].

The severity of TBI is commonly assessed using the Glasgow Coma Scale (GCS), which classifies injury as mild, moderate, or severe based on the patient’s level of consciousness following trauma [3]. Injury severity has been shown to be strongly associated with long-term neurological impairment, functional limitations, and increased healthcare utilization [4,5].

Although mortality rates associated with severe brain injury have declined over the past decades due to improvements in trauma care and neurocritical management, survival following moderate and severe TBI has increased. Consequently, a growing number of individuals are living with persistent physical, cognitive, emotional, and social consequences that significantly affect their long-term recovery and reintegration into society [6,7].

Beyond traditional clinical outcomes, quality of life (QoL) has emerged as a critical patient-centered indicator of recovery in individuals with TBI. QoL encompasses multiple dimensions of health, including physical functioning, psychological well-being, social participation, and perceived overall health status [8,9]. Previous studies have demonstrated that survivors of moderate and severe TBI frequently experience substantial reductions in QoL due to chronic symptoms, functional disability, and challenges in returning to work or maintaining social roles [10,11].

In addition to its clinical consequences, TBI also imposes a considerable socioeconomic burden on patients, families, and healthcare systems. Direct medical costs related to hospitalization, rehabilitation services, and long-term care are often accompanied by indirect costs such as productivity loss, changes in employment status, and household financial strain [12,13]. These economic pressures may further influence patients’ perceived well-being and recovery trajectory.

While several studies have examined the epidemiology and clinical outcomes of TBI, relatively limited evidence exists regarding the relationship between rehabilitation-related living costs and health-related QoL, particularly within Southern European healthcare settings [14,15]. Understanding how financial burden interacts with recovery outcomes may provide important insights for healthcare planning and rehabilitation policy.

The aim of this study was to investigate the longitudinal trajectory of QoL and to examine the association between household economic burden and QoL among patients with moderate and severe TBI during the first 12 months following hospital discharge. To the best of our knowledge, the findings presented in this study have not been previously published or presented elsewhere.

## Materials and methods

Study design and objectives

This study was designed as a prospective longitudinal observational study aiming to investigate the impact of living costs on the QoL of patients with moderate and severe TBI during the first 12 months following hospital discharge.

The primary objective was to assess the association between total living cost and QoL scores over time. Secondary objectives included the evaluation of QoL in patients with moderate and severe TBI, the measurement and comparison of living costs at baseline, six months, and 12 months post-discharge, and the examination of differences between moderate and severe TBI groups. No interventional procedures were performed as part of the study protocol.

Study setting and population

The study was conducted at the General Hospital of Attica “KAT,” a tertiary trauma referral center in Athens, Greece. A total of 100 patients aged 18 to 70 years who were hospitalized due to moderate or severe TBI were consecutively enrolled following hospital discharge.

Eligible participants were adults aged between 18 and 70 years with a confirmed diagnosis of moderate or severe TBI who had been discharged from hospital, were able to communicate in Greek, and provided written informed consent. Patients younger than 18 years, non-Greek speakers, individuals with severe pre-existing psychiatric or neurological disorders, and those unable to complete questionnaires without the availability of a proxy respondent were excluded from the study.

Classification of TBI severity

TBI severity was assessed upon admission using the GCS. Moderate TBI was defined as a GCS score between 9 and 12, while severe TBI was defined as a GCS score between 3 and 8. GCS scores were recorded by emergency department physicians during the initial clinical evaluation. The GCS was applied according to its standard clinical administration guidelines [13].

Data collection procedure

Participants were assessed at three predefined time points: at hospital discharge (baseline), at six months post-discharge, and at 12 months post-discharge. Data collection was conducted between June 2023 and June 2025. Questionnaires were administered either during scheduled follow-up visits or via structured telephone interviews when in-person assessment was not feasible. Telephone interviews were conducted using a standardized, structured format by trained members of the research team to ensure consistency in data collection across all participants.

Study Instruments

Living Cost Assessment

A structured cost-recording instrument was developed to quantify the economic burden associated with TBI (see Appendix A). The tool was designed to capture both direct and indirect costs related to injury and rehabilitation [14]. The questionnaire was developed based on previously published frameworks by Stergiannis (2012) and has been used in academic research. The original Greek version was used in this study without modification and with the permission of the original developer, who is also a co-author of the present study.

Direct medical costs included expenses related to medical consultations, nursing services, physiotherapy sessions, psychological support, social worker visits, medications, specialized medical supplies, hospital readmissions, and admissions to rehabilitation centers.

Direct non-medical costs included caregiver expenses, transportation expenses, special dietary requirements, home modifications, gym or rehabilitation program fees, and other injury-related expenditures.

Indirect costs included patient work absenteeism, occupational changes, income loss, and family members’ work absenteeism.

The total living cost per participant was calculated as the cumulative sum of all reported expenses for each assessment period.

The cost assessment instrument was developed specifically for the purposes of this study based on previously published frameworks of direct and indirect cost evaluation in TBI. The questionnaire consisted of structured items with predefined cost categories and was pilot-tested in a small group of patients prior to the main study to ensure clarity and completeness. Data collection followed a standardized protocol, and all participants were provided with the same instructions for reporting expenses.

QoL Assessment (SF-36)

QoL was assessed using the Short Form-36 Health Survey (SF-36), a validated instrument widely used to evaluate health-related QoL across multiple populations and clinical conditions (see Appendix B) [15,16]. The validated Greek version of the SF-36 was used in this study in accordance with the official administration and scoring guidelines [17]. The SF-36 was developed at RAND as part of the Medical Outcomes Study.

The SF-36 consists of 36 items grouped into eight domains: physical functioning, role physical, role emotional, social functioning, bodily pain, general health, vitality, and mental health. Each domain score ranges from 0 to 100, with higher scores indicating better perceived health status.

In the present study, the internal consistency reliability of the instrument was high (Cronbach’s α = 0.89).

Handling of missing data

Incomplete questionnaires were reviewed at the time of collection to minimize missing responses. For SF-36 scoring, if more than 50% of the items within a specific domain were completed, missing items were imputed using the mean of the completed items within that domain. If fewer than 50% of the items were completed, the domain score was treated as missing. Participants who were lost to follow-up were excluded from repeated-measures analysis but were included in baseline descriptive analyses.

Ethical considerations

The study was conducted in accordance with the Declaration of Helsinki. Ethical approval was obtained from the Ethics Committee of KAT General Hospital, Athens, Greece (approval no. E.S. 26016/11-05-2023). Written informed consent was obtained from all participants prior to enrollment. All participants provided consent for publication of anonymized data. Confidentiality and anonymity were strictly maintained, and data were used exclusively for research purposes in accordance with ethical guidelines.

Statistical analysis

Quantitative variables were presented as mean, standard deviation, median, and interquartile range, while categorical variables were expressed as absolute and relative frequencies. Normality of distribution was assessed using the Kolmogorov-Smirnov test.

For comparative analyses, the independent samples t-test was used for two-group comparisons, and one-way analysis of variance (ANOVA) was applied for multi-group comparisons. Repeated measures ANOVA was used for normally distributed repeated measurements, whereas the Friedman test and the Wilcoxon signed-rank test were applied for non-parametric repeated measures.

Correlation analysis was performed using Pearson’s correlation coefficient for normally distributed variables and Spearman’s correlation coefficient for non-normally distributed or ordinal variables.

Multiple linear regression analysis was conducted when the dependent variable was continuous and at least two statistically significant predictors emerged from bivariate analysis. Variables with p-values less than 0.20 in bivariate analysis were entered into the multivariate model, and backward elimination was applied to control for potential confounding effects. Regression coefficients, 95% confidence intervals, p-values, and R² values were reported. The level of statistical significance was set at α = 0.05.

All statistical analyses were performed using IBM SPSS Statistics for Windows, version 29.0 (released 2012, IBM Corp., Armonk, NY). All instruments used in this study were applied in accordance with their official administration and scoring guidelines.

## Results

A total of 100 patients were included in the study, with a mean age of 40.72 ± 10.9 years. Of these, 64% (n = 64) had moderate TBI, and 36% (n = 36) had severe injury (Table 1).

**Table 1:** Traumatic brain injury (TBI) level

		Frequency	Percent	Valid Percent	Cumulative percentage
Valid	Moderate	64	64.0	64.0	64.0
	Severe	36	36.0	36.0	100.0
	Total	100	100.0	100.0	

Demographic variables contained missing responses; therefore, percentages are reported based on valid responses for each variable. Marital status data were available for 85 participants. Among them, 58.8% (n = 50) were married, 25.9% (n = 22) were single, 12.9% (n = 11) were cohabiting, and 2.4% (n = 2) were divorced. Parental status data were available for 34 participants; of these, 91.2% (n = 31) reported having children, and 8.8% (n = 3) did not. The demographic characteristics of the study population are presented in Table 2.

**Table 2:** Demographic characteristics of the patients under study Percentages are calculated based on valid responses for each variable due to missing data.

		n	%
Age (n = 97)	20-30	19	19.6
	30-40	34	35.1
	>40	44	45.4
Marital status (n = 85)	Single	22	25.9
	Married	50	58.8
	Divorced	2	2.4
	Cohabiting	11	12.9
Education level (n = 75)	High School	12	12.9
	College	6	8
	University	20	26.7
	Postgraduate	37	49.3
Having children (n = 34)	Yes	31	91.2
	No	3	8.8

Most respondents (80.2%, n = 77; valid responses) reported living with others. Monthly family income data were available for 97 participants and were distributed as follows: 33.0% (n = 32) earned €1,001-1,500; 23.7% (n = 23) earned €2,001-2,500; 19.6% (n = 19) earned €1,501-2,000; 16.5% (n = 16) earned €501-1,000; and 7.2% (n = 7) earned €2,501-3,000. Employment status data were available for 97 participants; 85.6% (n = 83) were employed full-time. Employment sector data were available for 90 participants, of whom 61.1% (n = 55) worked in the private sector. Insurance provider data were available for 94 participants; the majority were insured under IKA (Greek Social Insurance Institute) (62.8%, n = 59) (Table 3).

**Table 3:** Economic and employment status of the patients under study

		n	%
Living with others (n = 96)	Υes	77	80.2
	Νo	19	19.8
Monthly family income (n = 97)	501-1000	16	16.5
	1001-1500	32	33.0
	1501-2000	19	19.6
	2001-2500	23	23.7
	2501-3000	7	7.2
Employment status (n = 97)	Full-time	83	85.6
	Part-time	7	7.2
	Retired	3	3.1
	Unemployed	4	4.1
Employment sector (n = 90)	Public	35	38.9
	Private	55	61.1
Insurance provider (n = 94)	IKA (Greek Social Insurance Institute)	59	62.8
	Public	32	34.0
	ΤΕΒΕ	2	2.1
	Οther	1	1.1

**Figure 1:**
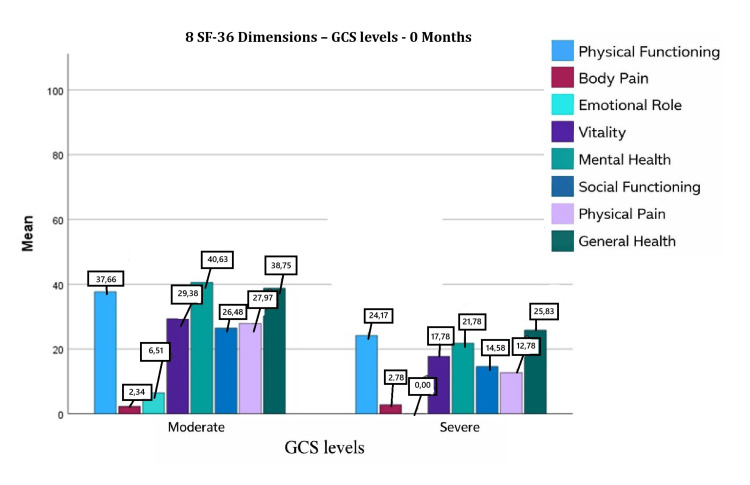
Eight SF-36 dimensions (GCS levels): 0 months

Immediately after discharge, all eight SF-36 dimensions were substantially below the normative baseline value of 50, with particularly low scores in role physical and role emotional. The overall SF-36 score was 21.77 ± 11.84 (median 22.11; interquartile range 17). Patients with moderate injury demonstrated higher scores than those with severe injury (26.01 ± 10.56 vs. 14.23 ± 10.18, respectively) (Figure 1 [15]).

At discharge, significant correlations were observed between SF-36 and marital status (r = −0.19, p = 0.02), educational level (r = 0.19, p = 0.02), monthly family income (r = 0.24, p < 0.01), and GCS score (r = −0.48, p < 0.001). In multiple linear regression analysis, GCS score was the only independent predictor, explaining 39% of the variance (R² = 0.39). Each one-unit increase in GCS score was associated with a 0.557-unit increase in SF-36 (p < 0.001).

**Figure 2:**
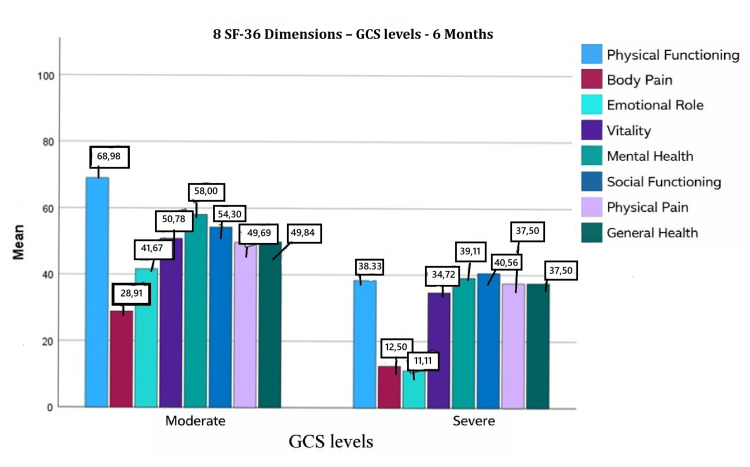
Eight SF-36 dimensions (GCS levels): six months

At six months, physical functioning, mental health, and general health exceeded the baseline value of 50. Vitality, social functioning, bodily pain, and the overall SF-36 score approached baseline levels, whereas role physical remained comparatively lower (Figure 2 [15]). The overall mean SF-36 score increased to 46.56 ± 19.10 (median 44.43; interquartile range 30). Patients with moderate injury continued to demonstrate higher scores than those with severe injury (54.76 ± 16.67 vs. 31.99 ± 13.77).

At six months, significant associations were identified between SF-36 and educational level (p = 0.006), employment sector (p = 0.02), monthly family income (p = 0.002), and GCS score (p < 0.001). Multiple regression analysis showed that both GCS score and monthly family income independently predicted SF-36. Each one-unit increase in GCS score increased SF-36 by 0.596 units (p < 0.001), and each increase in income category increased SF-36 by 0.164 units (p = 0.03). These variables explained 51% of the variance (R² = 0.51).

**Figure 3:**
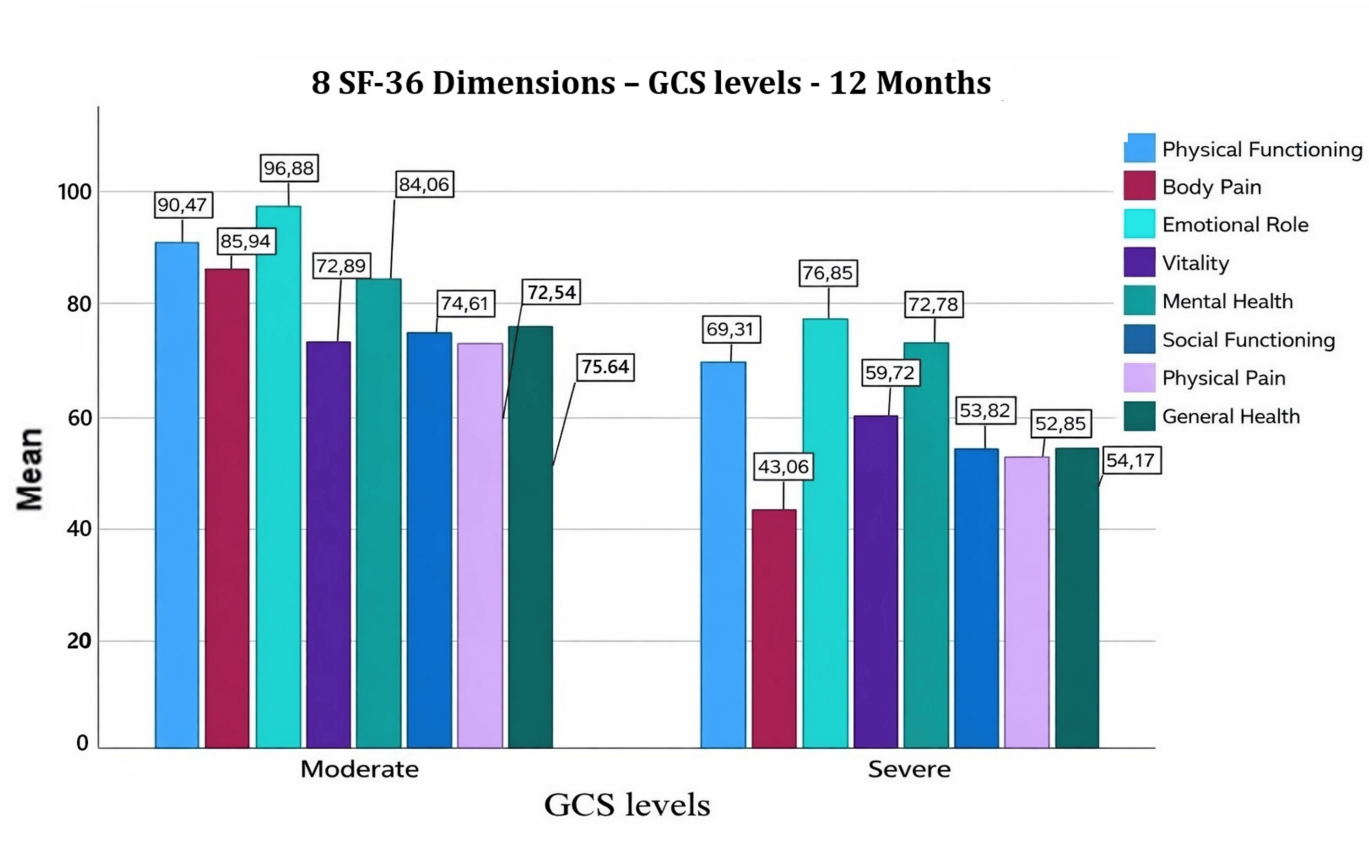
Eight SF-36 dimensions (GCS levels): 12 months

At 12 months, all SF-36 domains exceeded the baseline value of 50, with particularly high scores in physical functioning, role emotional, and mental health (Figure 3 [15]). The overall SF-36 score reached 70.71 ± 15.51 (median 73.03; interquartile range 20). Mean values remained significantly higher in patients with moderate injury compared to those with severe injury (78.55 ± 9.56 vs. 56.78 ± 14.30; p < 0.001).

In multiple regression analysis at 12 months, GCS score positively predicted SF-36 (b = 0.683, p < 0.001), whereas the number of children negatively predicted SF-36 (b = −0.247, p < 0.001). These variables explained 63% of the variance (R² = 0.63).

A statistically significant improvement in QoL over time was observed (F = 198.02, p < 0.001). Mean SF-36 scores increased from 21.77 ± 11.83 at discharge to 46.56 ± 19.10 at six months and 70.71 ± 15.51 at 12 months (Table 4). Improvement was significantly greater in patients with moderate injury compared to those with severe injury (p < 0.001).

**Table 4:** SF-36 domain scores across follow-up assessments SF-36 domain scores of participants at discharge, six months, and 12 months after hospitalization. Higher scores indicate better health-related quality of life (Short Form-36 Health Survey) [16,17].

	Mean SF-36	SD
0 months	21.77	11.83
6 months	46.56	19.10
12 months	70.71	15.51

The mean total rehabilitation cost at 12 months was €7,134.50 ± 3,981.35 (median €6,040.00). The mean household income loss due to work absence was €1,957.50 ± 3,183.30 (median €500.00). The overall mean total household burden was €9,092.00 ± 5,634.15 (median €8,160.00).

At discharge, the mean rehabilitation cost was €1,019.90 ± 1,470.89 (median €520.00). A significant negative correlation was observed between rehabilitation cost and SF-36 (r = −0.31, p < 0.001). Mental health, vitality, bodily pain, and general health were significantly negatively correlated with cost. Multiple regression analysis showed that employment sector and GCS score explained 18% of cost variance (R² = 0.18). Private-sector employment was associated with higher costs (p = 0.02), while higher GCS scores were associated with lower costs (p = 0.001).

**Figure 4:**
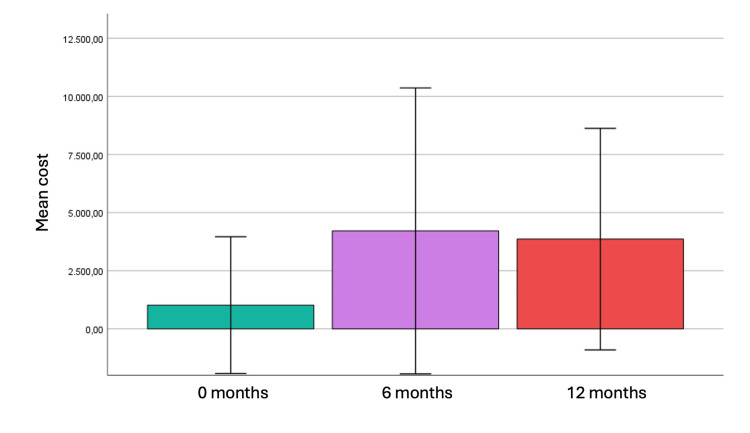
Increase in rehabilitation cost over time

During the first six months, the mean rehabilitation cost was €4,212.60 ± 307.40 (median €3,575.00). Rehabilitation costs increased significantly over time (p < 0.001) (Figure 4). Costs were significantly higher in patients with severe injury, and a strong negative association was observed between GCS score and cost (p < 0.001). An inverse relationship between rehabilitation cost and SF-36 was again identified, indicating that greater economic burden was associated with poorer QoL.

## Discussion

A key strength of this study is its prospective longitudinal design with repeated follow-up assessments, allowing for a detailed evaluation of recovery trajectories over time.

The present study investigated the longitudinal progression of QoL and its association with demographic, socioeconomic characteristics, and injury severity among patients with moderate and severe TBI at discharge, six months, and 12 months post-hospitalization.

Quality of life trajectory

The findings demonstrate a consistent and statistically significant improvement across all eight SF-36 dimensions over the 12-month follow-up period. QoL scores were markedly reduced at discharge, particularly in role physical, role emotional, social functioning, and bodily pain. Substantial recovery was observed at six months, with further pronounced improvement by 12 months.

By 12 months, all SF-36 domains exceeded the normative baseline score of 50, suggesting clinically meaningful recovery. Physical functioning, role, emotional, and mental health achieved the highest levels, indicating significant functional and psychological restoration. These results suggest that QoL following TBI improves progressively rather than remaining static, highlighting the importance of sustained rehabilitation and long-term follow-up care [4,5].

Influence of injury severity

Across all time points, patients with severe TBI consistently exhibited significantly lower QoL scores compared to those with moderate injury. GCS score emerged as the strongest independent predictor of QoL in all regression models. The effect of GCS increased over time, indicating that initial neurological severity continues to influence long-term functional outcomes. This finding aligns with previous research demonstrating that lower GCS scores are associated with prolonged disability and reduced health-related QoL [4].

Sociodemographic factors and QoL

Monthly family income demonstrated a significant positive association with QoL, particularly at six months. Financial stability may facilitate access to rehabilitation services, supportive care, and psychological resources, thereby enhancing recovery. Although the effect diminished slightly by 12 months, income remained an important determinant of outcome.

Higher educational attainment was also associated with improved QoL at later time points. Education may function as a proxy for health literacy, socioeconomic resources, or cognitive reserve, all of which may contribute to adaptive coping and rehabilitation engagement.

Marital status and living with others were modestly associated with better QoL in earlier recovery phases, suggesting a protective role of social support during the acute and subacute rehabilitation period.

At 12 months, the number of children was negatively associated with QoL and emerged as an independent predictor in the regression model. Greater family responsibilities may intensify emotional and financial stress, thereby influencing perceived well-being during long-term recovery.

Economic burden and quality of life

The analysis of rehabilitation-related costs revealed a substantial financial burden over the 12-month period, particularly among patients with severe TBI. Costs increased significantly over time, reflecting ongoing rehabilitation needs.

Higher income levels were associated with greater rehabilitation expenditure, likely reflecting improved access to private or supplementary services. Importantly, increased economic burden was consistently associated with lower QoL, particularly in domains related to mental health, vitality, social functioning, and general health. These findings are consistent with previous literature emphasizing the economic impact of TBI on long-term outcomes [6,7].

Evolution of functional domains

Domain-specific analysis revealed differential recovery patterns. General health, vitality, bodily pain, and mental health demonstrated steady and sustained improvement. Social functioning, although severely impaired at discharge, showed gradual recovery over time. Role physical exhibited marked improvement between six and 12 months, suggesting delayed but meaningful functional gains. The role of emotion, initially profoundly reduced, demonstrated substantial recovery by 12 months.

These patterns underscore the non-linear nature of TBI recovery, with certain dimensions improving rapidly while others require prolonged rehabilitation efforts.

Limitations

This study has several limitations. First, the single-center design may limit the generalizability of the findings to other healthcare settings or populations. Second, rehabilitation and household cost data were self-reported and therefore subject to recall bias and potential reporting inaccuracies. Third, the cost assessment tool, although structured, was specifically developed for the purposes of this study and has not been externally validated, which may limit full reproducibility. Additionally, pre-injury QoL was not assessed, preventing direct comparison with baseline status prior to trauma. Finally, the relatively modest sample size, although adequate for exploratory analysis, may limit the statistical power and robustness of multivariate modeling. Future multicenter studies with larger sample sizes and standardized economic assessment tools are warranted to validate and extend these findings.

## Conclusions

This study demonstrates that QoL in patients with moderate and severe TBI improves significantly over the first 12 months following hospital discharge; however, recovery is strongly influenced by both injury severity and socioeconomic factors. GCS score emerged as the most consistent predictor of long-term outcomes, while financial resources played a significant role in shaping rehabilitation access and overall well-being.

The findings emphasize that TBI is not solely a neurological condition but also a socioeconomic challenge. Comprehensive rehabilitation strategies should therefore integrate both medical and financial support mechanisms in order to optimize long-term recovery and QoL.

## Figures and Tables

**Figure 5:**
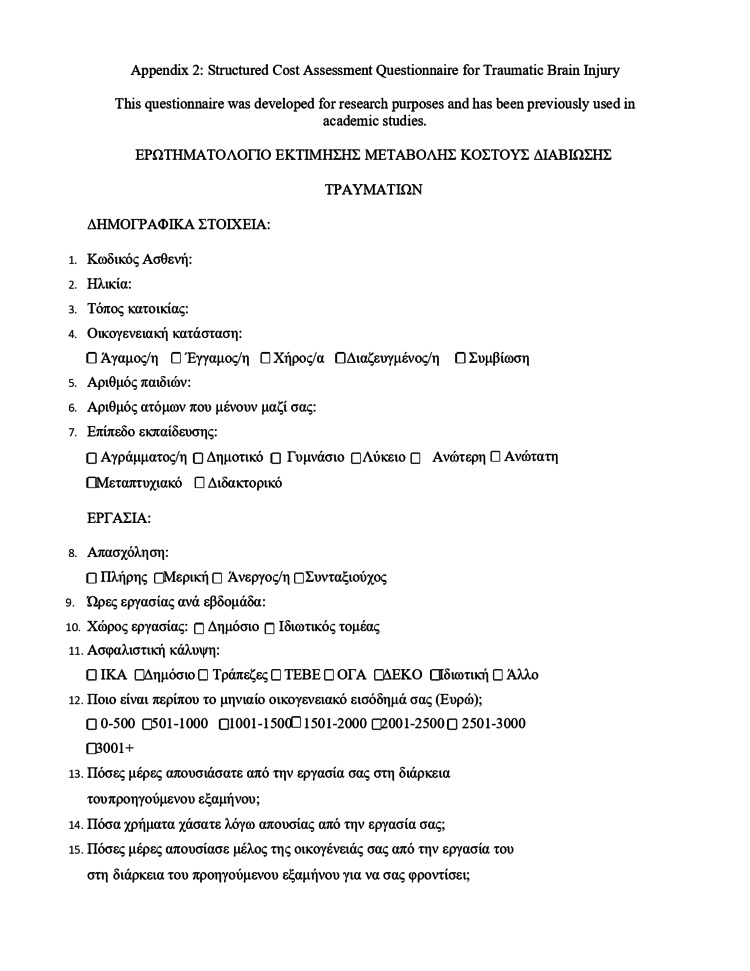
Structured Cost Assessment Questionnaire (page 1)

**Figure 6:**
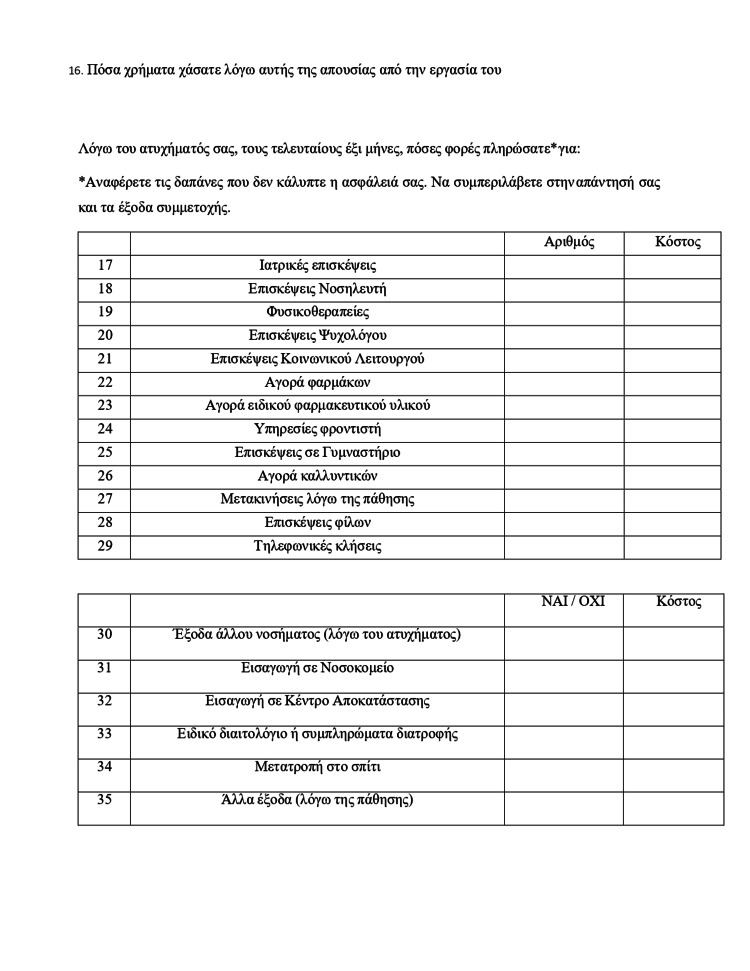
Structured Cost Assessment Questionnaire (page 2)

**Figure 7:**
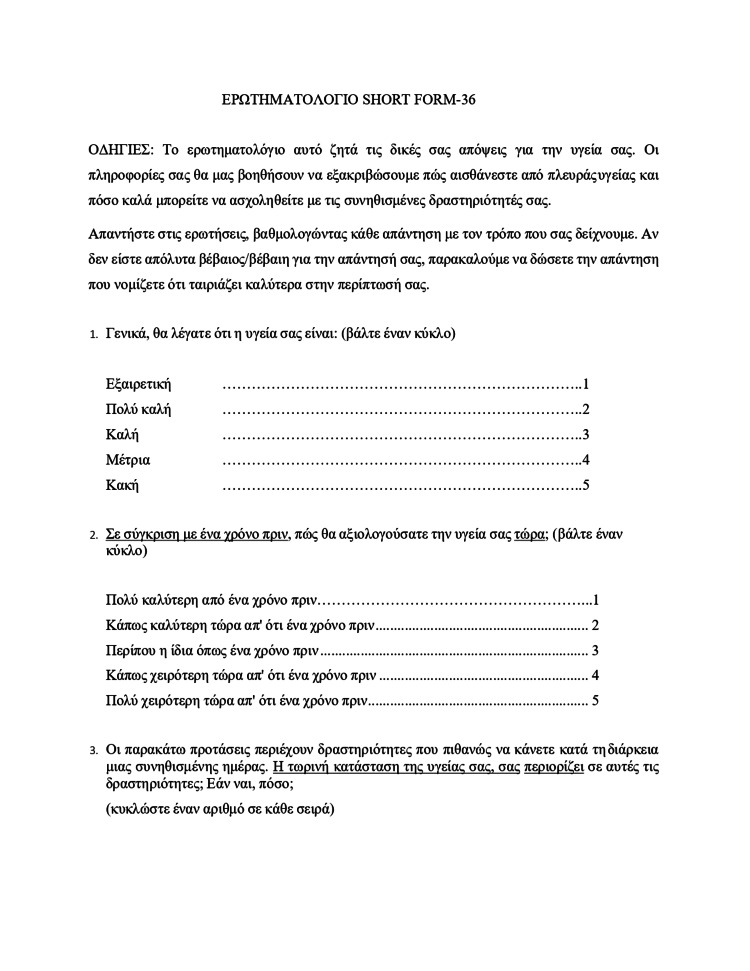
Short Form-36 (Greek version) (page 1)

**Figure 8:**
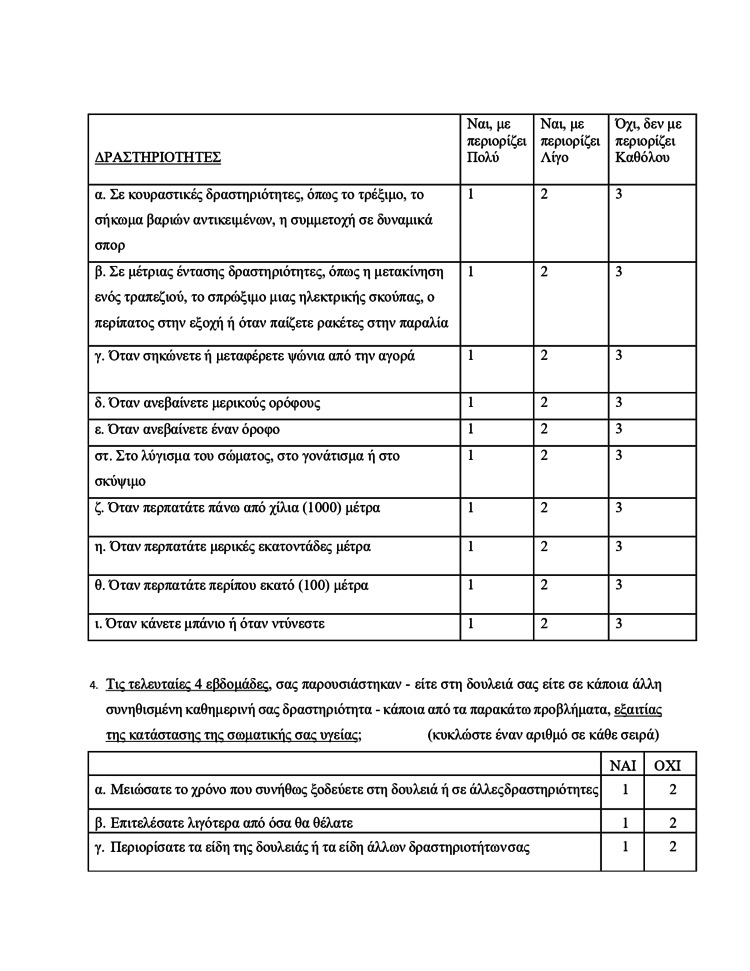
Short Form-36 (Greek version) (page 2)

**Figure 9:**
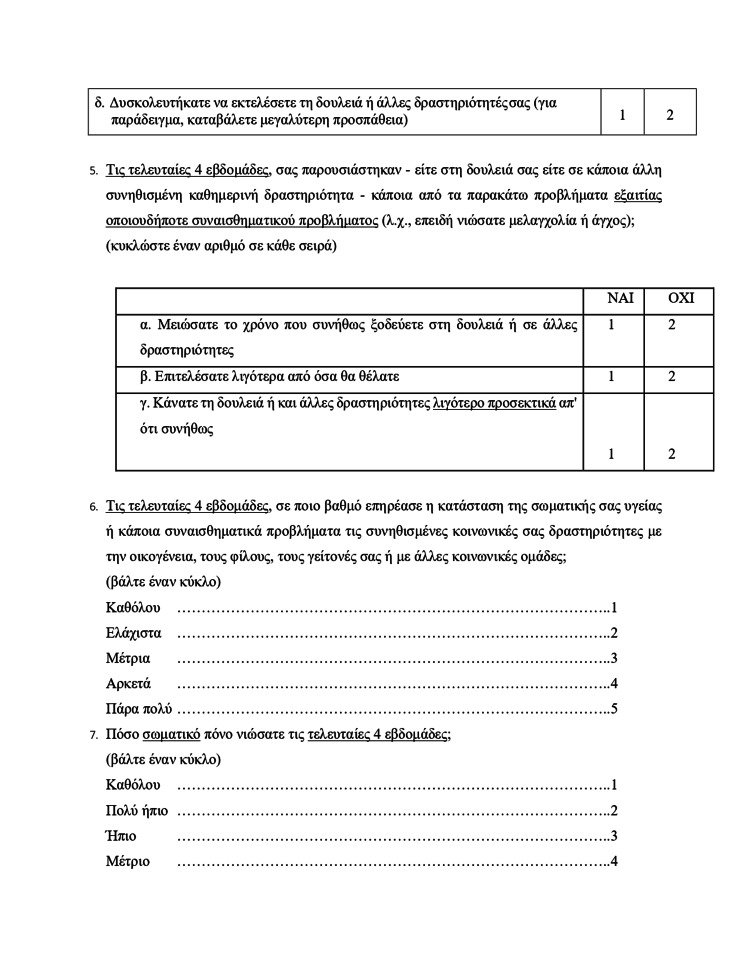
Short Form-36 (Greek version) (page 3)

**Figure 10:**
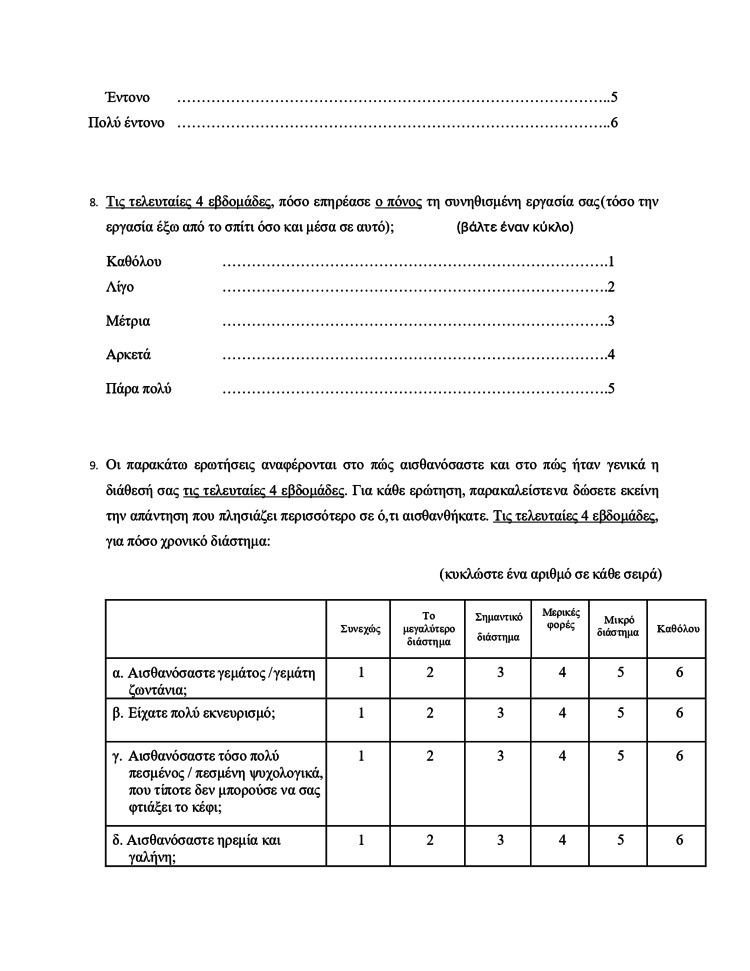
Short Form-36 (Greek version) (page 4)

**Figure 11:**
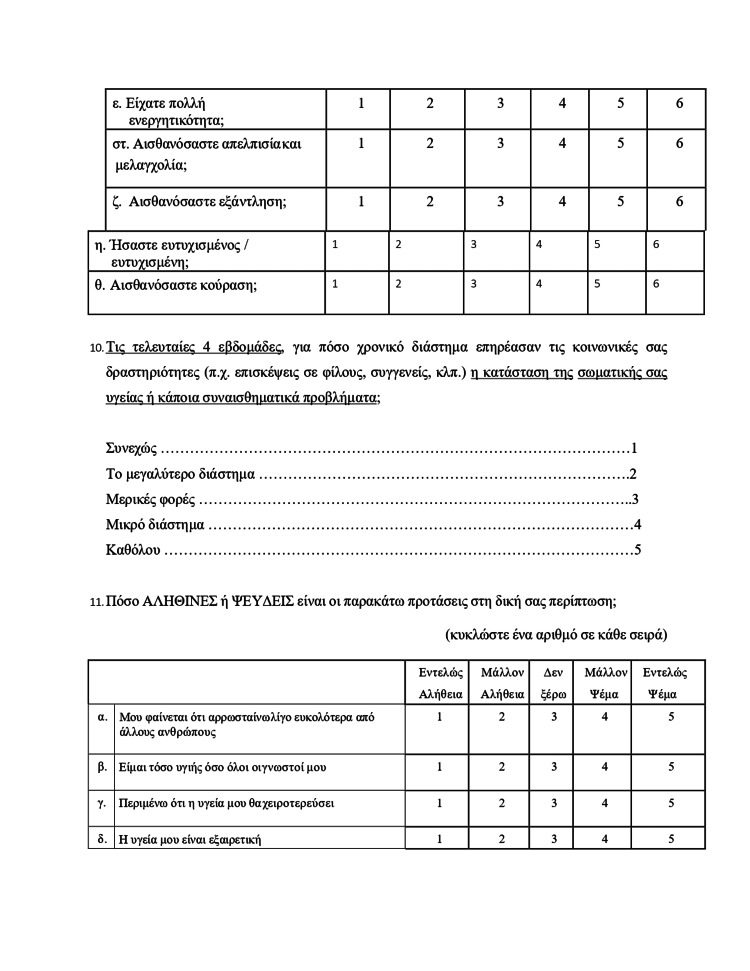
Short Form-36 (Greek version) (page 5)

## Appendices

Appendix A 

Appendix B
